# Microplastics in the European native oyster, *Ostrea edulis*, to monitoring pollution-related patterns in the Solent region (United Kingdom)

**DOI:** 10.1007/s10661-025-13975-x

**Published:** 2025-04-12

**Authors:** Lina M. Zapata-Restrepo, Katherine Bawden, Giovanna Sidaoui-Haddad, Eleanor Spencer, Ian D. Williams, Malcolm Hudson

**Affiliations:** 1 School of Geography and Environmental Sciences, University of Southampton, Highfield Campus, University Road, Southampton, SO17 1BJ UK; 2 Institute of Biology, Faculty of Exact and Natural Sciences, University of Antioquia, Medellín, Colombia; 3 School of Engineering, Faculty of Engineering and Physical Sciences, University of Southampton, Highfield Campus, Southampton, SO17 1BJ UK

**Keywords:** Oysters, Pollution, Bioaccumulation, Biomonitoring, Microplastics

## Abstract

Microplastics (MPs) are the most abundant type of debris in the marine environment, creating a significant threat to aquatic ecosystems due to their persistence, ability to absorb organic pollutants and potential ingestion by marine fauna. Shellfish are particularly vulnerable to MPs accumulation as they filter large volumes of seawater, and they become an important route for human exposure to these particles. This study, the first to examine MPs in European flat oyster (*Ostrea edulis*) populations, aimed to quantify these particles in the gill and digestive tissues of oysters from the Solent region (southern England). Enzymatic digestion using Proteinase-K was used in this study and MPs were identified in every oyster sampled to determine whether differences in abundance, type and size of MPs exist between locations. Oysters near urban areas contained significantly more MPs than those near rural areas. Fibres were the most prevalent type of MPs, with sizes varying across locations. The study found no significant differences in MPs presence between gills and digestive tissues, and an inverse correlation between the size of MPs and oyster size. The presence of MPs in wild *O*. *edulis* could be an additional threat to the survival of an already threatened species and may pose health risks for predatory species and human consumers of seafood. The use of *O*. *edulis* as a biomonitoring species for marine MPs pollution could help determine the extent, distribution and sources of MPs, potentially informing management strategies to reduce pollution.

**Supplementary Information:**

The online version contains supplementary material available at 10.1007/s10661-025-13975-x.

## Introduction

Worldwide plastic production has increased considerably, from 1.5 million tonnes in 1950 to 391 million tonnes in 2021 (PlasticsEurope, 2022). The presence of plastics in the marine environment is considered by scientists to be a hazardous waste and a long-term threat to aquatic ecosystems (Avio et al., 2017; Cole et al., 2011; Law & Thompson, 2014; Moore, 2008). Recognised as the most abundant type of plastic waste, microplastics (MPs), defined as fragments or particles of plastic < 5 mm (ISO/TR 21960, 2020) are considered to be a particular cause for global concern and of local relevance.

MPs may have different impacts on organisms in comparison to larger plastic debris (Law & Thompson, 2014). Indiscriminate feeders at lower-trophic level present limited abilities to differentiate between plastic particles and food making them particularly susceptible to ingesting MPs (Wright, Thompson, & Galloway, 2013b). For instance, they can be ingested by a wide range of organisms while feeding, including marine fauna at lower trophic levels such as planktonic organisms, echninoderms, polychaetes and bivalves (Cole et al., 2013; Graham & Thompson, 2009; Van Cauwenberghe et al., 2015).

Due to their extensive filter-feeding activity, bivalves are vulnerable to the ingestion of MPs (Cho et al., 2019; Li et al., 2015; Naidu, 2019) and partially attributable to the feeding and ventilation mechanisms of the gills (Green, 2016). These organisms are capable of sorting particles prior to ingestion, selecting them based on their size, shape and density (Ward & Shumway, 2004; Ward et al., 2019). However, the nutritional value of the particles can be mistaken by ingesting MPs adsorbed to organisms (Wright et al., 2013b). The ingested MPs can be taken up by epithelial cells in the intestinal tract and even translocate through the intestine wall into the circulatory system (Browne et al., 2008). This can result in limited food uptake through the blockage of feeding appendages and the alimentary canal (Van Cauwenberghe et al., 2015; Von Moos et al., 2012).

A range of effects has been reported in bivalves after microplastic ingestion (Baroja et al., 2021; Khanjani et al., 2023), including inflammatory response in gastrointestinal tract and cells of the digestive system (Von Moos et al., 2012), translocation into circulatory system (Browne et al., 2008), changes in respiration rates (Green, 2016), reduction in feeding, gamete quality and fecundity (Sussarellu et al., 2016). There are indirect impacts caused by MPs in the marine environment, including the ability of MPs to adsorb a range of persistent organic pollutants (POPs) onto their surface, such as polychlorinated biphenyls (PBCs), which can cause toxic effects (Wang et al., 2018).

The potential for persistence and accumulation of microplastic particles in oysters presents implications for their predators, including birds, crabs and fish (Wright et al., 2013b). With an increasing demand for fish and shellfish globally, and the trophic transfer of MPs along the aquatic food chain, bivalves are considered to be an important route of human exposure to MPs because they filter a large volume of seawater while feeding and, thus accumulate MPs from seawater (Van Cauwenberghe & Janssen, 2014). The potential impact on human health through trophic chain is possible but not (yet) clear (Anbumani & Kakkar, 2018; Van Cauwenberghe & Janssen, 2014; Wang et al., 2019).

Bivalves are of particular interest due to their importance in benthic assemblages and their high economic value in the seafood industry worldwide; and their extensive filter-feeding activity, which directly exposes them to MPs present in the water column (Browne et al., 2008; Van Cauwenberghe & Janssen, 2014). Bivalves have proved to be useful bioindicators for environmental pollution and improve understanding of the relationship between human health and exposure to environmental contaminants. *Ostrea edulis*, the largest oyster native to Europe is a bivalve species of family Ostreidae found from the low intertidal down to the sublittoral zone throughout the Atlantic and Mediterranean coasts of Europe (CABI, 2019; Perry & Jackson, 2017). It is recorded at higher abundance on the Mediterranean coasts of Europe especially in association with highly productive estuarine areas (Gibson et al., 2007; Harding et al., 2016; Laing et al., 2005). *O*. *edulis* populations are valuable both ecologically and economically, and today the species is the focus of concerted restoration efforts across Europe and beyond (Bennema et al., 2020).

England’s Solent Strait holds considerable ecological and scenic value, with many zones designated internationally for protection and forming parts of nationally important protected landscapes. However, this semi-enclosed waterway is frequently used by commercial, naval and public transport vessels that contribute to a degradation of water quality in this area (Xiong et al., 2023). Furthermore, the Solent Strait is bordered by agricultural lands and urban areas, including wastewater treatment plants (WWTPs), which also affect the marine water quality (Solent Forum, 2022).

In this study, wild populations of *O*. *eduli*s in the Solent region were examined to determine the quantities of MPs within individual oysters. *O*. *edulis* was chosen as the study species because, as of yet, there has been no research investigating the presence of MPs in *O*. *edulis* in the area and this species is already classified as a threatened species (OSPAR, 2008). The life characteristics of this species such as a wide geographic range, sessile nature, basal position in the food web, accessible habitat in shallow estuarine waters, easy cultivation as well as its well understood biology make it a suitable candidate for environmental monitoring. Being a sessile suspension feeder, oysters can effectively reflect ambient environmental contamination. Hence, we assumed that domestic and industrial activities are important sources of MPs in the Solent area and expected relatively high concentrations of MPs in *O*. *edulis* tissues with some differences between locations. Gills and digestive tract have been reported as important tissues in the translocation process of these particles once they enter onto the organisms, thus the differential presence of MPs in gill and digestive tissues was expected. The exposure of this species to MPs can reflect an ecological and human risk in the Solent. Monitoring the uptake of MPs into this species could serve as a gauge for other marine invertebrate species and provide useful information concerning the levels of microplastic pollution in the Solent.

## Methods

### Study area and sampling

**Fig. 1:**
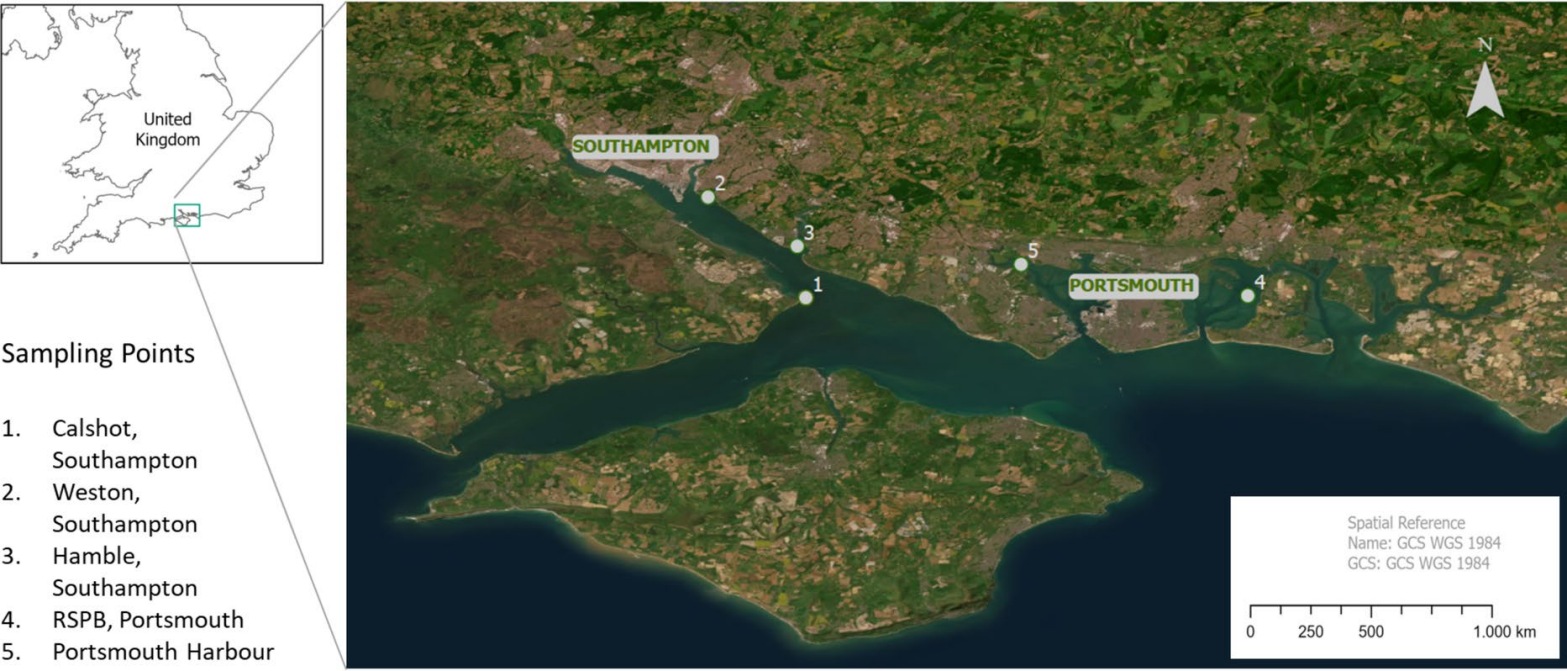
*Ostrea edulis* sampling locations in the Solent region including Southampton and Portsmouth areas

*O*. *edulis* were collected from July to November 2016 from the following intertidal locations within the Solent region: Calshot, Weston, Hamble estuary, Langstone Royal Society for the Protection of Birds (RSPB) Reserve in Portsmouth and the Portsmouth Harbour (Fig. 1). The locations were located based on associated different anthropogenic activities (Table 1).

**Table 1:** Characteristics of each sampling point and number of oysters collected per location

Location	Coordinates	Oysters collected (*n*)	Characteristics
Calshot, Southampton	50.816272, − 1.306495	4	Relatively low levels of chemical pollutants Greater tidal mixing (Williams & Muxagata, 2006)
Weston, Southampton	50.886546, − 1.375198	5	Site of Special Scientific Interest (SSSI) (Mouchel, 2012). Residential properties and park-land around the area (Mouchel, 2012). Proximity to the pollutant inputs from storm-water discharges, Woolston WWTP and other industrial contaminants from the River Itchen (Gallagher et al., 2016; Yates, 2015)
Hamble, Southampton	50.852299, − 1.3125556	8	A major yachting centre in the Solent; an important centre for recreational boating; shielded from the open sea by the Isle of Wight (McAuliffe et al., 2014)
Langstone Royal Society for the Protection of Birds (RSPB), Portsmouth	50.817642, − 1.008355	6	A semi-enclosed tidal inlet area with poor flushing (Soulsby et al., 1978); receives significant sewage and frequent storm-water discharges, particularly from Budds Farm (Lockwood et al., 1985)
Portsmouth Harbour	50.83955, − 1.15525	4	Large industrialized estuary; Ramsar site; Special Protection Area; a Biological Site of Scientific Interest in its proximity

Between four and eight oysters were collected from coastal locations in southern England and transported to the laboratory where biological data (such as shell length, width, height and weight) were measured. Oysters were immediately stored at – 20 °C (Oaten et al., 2015). Prior digestion, defrosted soft tissues were removed from shells, rinsed with commercial ultrapure water (HPLC grade, Daejung Chemicals and Metals Co.) to remove remaining fine shell particles and external contaminants and placed in glass containers. A mass of 0.2 g of gill and digestive tract tissue from each oyster was dissected and weighed and placed in 100 mL glass containers and sealed immediately with metal screw top lids. Glassware was soaked for several hours in acid water (a 10% solution of hydrochloric), then rinse with distilled water and dry before proceeding. Two replicates for each tissue type were obtained and placed in separate containers. Dissection tools (razor blade and dissection tweezers) were washed thoroughly with ultrapure, deionised water (MilliQ) between replicates and samples to prevent cross-contamination (Bogdanowicz et al., 2021; Claessens et al., 2013). Animals used throughout this project (Project ID: 20658) were kept and manipulated according to the ethics guidelines and under the approval of the Ethics and Research Governance Online system (ERGO) of the University of Southampton (Faculty of Environmental and Life Science Ethics Committee) under the University’s Ethics and Research Governance Policy.

### Digestion

An enzymatic digestion method adapted by Cole and colleagues (Cole et al., 2014) and optimized by Karlsson and colleagues (Karlsson et al., 2017) for analysis of MPs in biota was followed with brief modifications. After adding 15 mL homogenisation buffer (400 mM Trizma-HCL, 60 mM EDTA, 150 mM NaCl, 1% SDS, pH 8), a glass-on-glass manual-powered Dounce homogenizer (Bellco Glass) as used to manually break down the tissue. After a 15-min incubation period, 8 mg of Proteinase-K (3.0–15.0 unit/mg, *T*. *album*, Sigma-Aldrich) was added to each container and incubated for 2 h at 50 °C (Catarino et al., 2016; Cole et al., 2014). Sodium perchlorate (375 µL) was then added to aid deproteinization (Wilcockson, 1973). The samples were placed on a shaking table at room temperature for 20 min before being incubated at 60 °C for a further 20 min. The samples were vacuum filtered through a Whatman GF/C filter (1.2 μm, ⌀ 47 mm) and rinsed with > 100 mL MilliQ water. The filters were then covered and dried in the oven at 60 °C overnight. Once dried, filters were stored in aluminium lined petri dishes.

### Identification

All the filters were inspected under an Olympus BH2 polarised light microscope (magnification × 40), and photographic images were obtained using a Nikon D5000 digital camera. Following the microplastic classification method by Kyong Song et al. (2015) and Gallagher et al. (2016), each particle was photographied and categorised into one of three main types; fibre, irregular or round (including oval shapes). Also, the colour of each particle was recorded and some internal criteria were assumed in order to standardize categories (Table S1). ImageJ was used to determine the size of the particles (length was reported for fibres, widest point for irregular particles and diameter for round particles) (Ferreira & Rasband, 2015).

Additionally, Raman spectroscopy was performed using a 785 nm Reinshaw inVita with Leica DM 2500 M microscope, 50 × magnification lens. Approximately 1/3 of each filter was systematically analysed due to high particle loads as suggested by Schymanski et al. (2020). This analysis included the most common particles found (i.e. white/transparent, blue, grey and pink fibres) across the samples. Extended spectra were obtained at 1% of the total laser power to avoid melting of the particles, and if needed the intensity was increased. Then, 10–30 s exposure time and three accumulations were used. WiRE 4.1 software was used to process the data. All spectra were pre-processed in WiRe 4.1 by removing cosmic rays (if present), subtracting the baseline and smoothing them. BioRad KnowItAll was used to identify the Raman spectra by individual and multi-components, which were compared to a personal spectral library by Thiele (2019) and the Spectral Library of Plastic Particles aged in the environment (SLoPP) Raman library, both especially developed for MPs research (Munno et al., 2020).

### Contamination and control measures

During all sample treatment and analysis steps, precautions were taken to avoid background plastic contamination. Procedures were carried out in a clean fume hood. While carrying out tissue extractions and handling samples, a 100% cotton lab coat was worn at all times (Van Cauwenberghe & Janssen, 2014). To prevent contamination, all solutions were filtered through glass fibre filters (GF/F 0.7 mm, Whatman, Maidstone, United Kingdom (UK)) before use. Glassware was used instead of plasticware wherever possible. All glassware used in the digestion and filtration procedures was acid washed overnight, rinsed thoroughly in MilliQ® water and covered in aluminium foil and dried in an oven at 60 °C. All equipment was thoroughly rinsed with MilliQ® water (Millipore Corporation) before usage. All equipment and glassware were then covered with aluminium foil until use. Dissection tools used were washed thoroughly with ultra pure, deionised water (MilliQ) between replicates and samples to prevent cross-contamination. Samples and filters were covered with aluminium foil at every possible stage in the procedure to minimize exposure to airborne particles. Procedural blanks were measured with one blank per five sample analyses (Bogdanowicz et al., 2021). The average blanks, for each set of samples, were then subtracted from the particle count in each field sample to correct for background contamination.

Recovery efficiencies were calculated using the most typical MPs reported in environmental samples such as high-density polyethylene (HDPE), polyvinyl chloride (PVC), polyethylene terephthalate (PET) and expanded polystyrene (EPS) (Claessens et al., 2011). Small fragments were created from different commercial materials and polymer type was confirmed using attenuated total reflectance Fourier-transform infrared spectroscopy (ATR FT-IR) (Frontier, Perkin Elmer) with spectrum infrared spectroscopy software (Perkin Elmer). Known quantities of these particles were added to oyster tissues, and the same protocol was consistently applied to all samples. Microplastic spikes were chosen with distinct characteristics making identification and separation from contamination sources possible. The method showed a recovery rate of 90%, 85%, 80% and 100% for HDPE, PVC, PET and EPS, respectively.

### Data and statistical analyses

Wet tissue were used for all the calculations. The count of MPs was determined based on the particle categories confirmed to be plastic; particles from categories that were not assessed were excluded from the analysis (Horton et al., 2017). Microscopic inspection showed a total of 2197 particles, including fibres, irregular and round particles (Fig. S1), from gills and digestive tissues from oysters analysed in this study. For example, cotton was the predominant material (20%) detected in the oyster samples (Fig. S2). After subtracting the particles identified as cotton fibres, a total of 1751 particles were considered for the analysis. Linear mixed effect models (LMERs) were used in order to determine whether there were significant independent effects or interactive effects of location and MPs count, type and size using the lmer package (Bates et al., 2015). This was done using the lme4 package in R (version 4.3.2). For this a lmer was first fit to the data incorporating all results to see whether responses could be generalised. For each lmer residuals were checked for normality and homoscedasticity using the Shapiro–Wilk test and Levene’s test. As the data had a non-normal distribution they were Log transformed. Then, to compare means between mean counts of the gill and digestive tissue and the different types of MPs a Tukey’s Honestly Significance Difference (HSD) post hoc test was performed using SPSS v29. For the above statistical tests, a significance level of 0.05 was used.

## Results

### Identification and characterisation of particles

Blank controls showed low values of particles for all the locations. Fibres were the most common particle found in blanks with an average of 3, 8, 10, 6 and 4 for Calshot, Weston, Hamble, Posrtmourht Harbour and Langstone, respectively. One irregular particle was found in blanks from Calshot, Hamble, and Langstone; just one round particle was found in blanks from Calshot.

A subsample of 183 particles was analysed with Raman spectroscopy. Acrylic was the dominant polymer (32%), followed by rayon (22%) and cotton (18%). Only hits with a hit quality index (HQI) higher than 70 were accepted in this study (*n* = 152) (Fig. S1). The HQI value represents a numerical measure of the closeness of fit between an unknown spectrum and the reference one (Banik and Nedwed, 2005). The highest and most consistent HQI scores throughout all the samples corresponded to the reference spectrum of the GFC fibre.

### Microplastics count in *O. edulis*

**Fig. 2:**
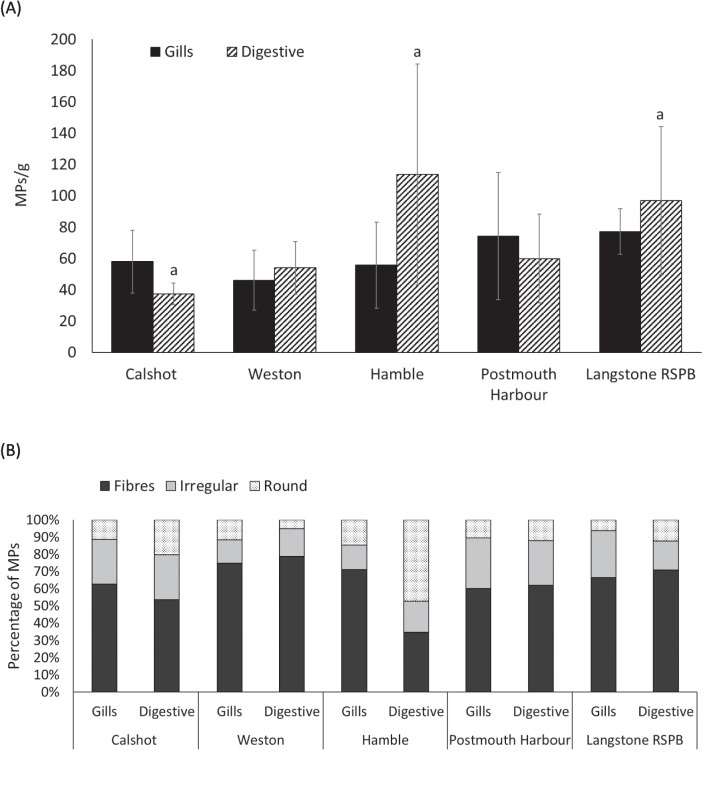
**A** The concentration of MPs per gram of tissue of *Ostrea edulis* and **B** percentage of each type of particle found observed in gill and digestive tissues at Calshot (*n* = 4), Weston (*n* = 5), Hamble (*n* = 8), Portsmouth Harbour (*n* = 4) and Langstone RSPB (*n* = 6). Same letters represent significant differences (*p* < 0.05). Error bars represent S.E

Fibres, irregular and round MPs were observed on the filters of the *O*. *edulis* tissue extracts (Supplement, Fig. 1). The most common type of particles were fibres (1046), followed by irregular (356) and round particles (348). The MPs concentrations (mean ± S.E.) showed different behaviour depending on the tissue. The analysis of MPs per gram of wet digestive tissue in oysters showed the highest levels in Hamble (113.70 ± 70.48), followed by Langstone RSPB (96.88 ± 47.24), Portsmouth Harbour (59.87 ± 28.38), Weston (54.04 ± 16.96) and Calshot (37.36 ± 7.01), which had the lowest mean microplastic counts in that tissue (Fig. 2A). The MPs concentrations found in wet tissue of gills of oysters collected from all locations were relatively similar. Langstone RSPB exhibited the highest concentration (77.29 ± 14.37), followed by Portsmouth Harbour (74.28 ± 40.50), Calshot (58.07 ± 20.05), Hamble (55.78 ± 27.58) and Weston (46.17 ± 19.26), which had the lowest concentration.

The analysis of MPs found in both tissues reveals a general pattern in the abundance of microplastic types, with fibres accounting for the largest proportion of the mean microplastic count across all locations. Significant differences were observed for fibres in Hamble (*p* < 0.001) and Langstone (*p* = 0.001) (Table 2, Fig. 2B). Results from the LMERs indicate a significant interactive effect of location and MP concentrations. Specifically, oysters from Langstone showed a significant difference in fibre abundance compared to those from Calshot (*p* = 0.00163) and Hamble (*p* = 0.0448). Additionally, oysters from Weston exhibited significant differences in the abundance of irregular particles compared to individuals from Langstone (*p* < 0.001) and Portsmouth Harbour (*p* = 0.0046). For round particles, oysters from Weston also differed significantly from those collected in Hamble (*p* = 0.0388). While fibres consistently represent the most abundant MP type, the abundance of irregular and round particles is generally lower. An exception was observed in oysters from Hamble, where the digestive tissue showed a higher mean count of round particles compared to other locations.

**Table 2:** The MPs mean ± S.E. per animal, categorized by shape (fibres, irregular or round), in gill and digestive tissues across different locations in the Solent region

Location	Oysters	Tissue	Number of MPs per tissue	MPs average per animal (mean ± S.E.)		
				**Fibres**	**Irregular**	**Round**
**Calshot**	*n* = 4	Gills	114	9.0 ± 3.2	3.8 ± 0.7	1.6 ± 0.7
		Digestive	69	4.6 ± 1.5	2.3 ± 0.3	1.8 ± 0.6
**Weston**	*n* = 5	Gills	103	7.7 ± 1.1	1.4 ± 0.4	1.2 ± 0.6
		Digestive	118	9.3 ± 2.1	1.9 ± 0.4	0.6 ± 0.2
**Hamble**	*n* = 8	Gills	204	9.1 ± 1.5	1.8 ± 0.4	1.9 ± 0.5
		Digestive	422	9.1 ± 2.2	4.8 ± 1.3	12.5 ± 4.6
**Portsmouth Harbour**	*n* = 4	Gills	133	10.0 ± 2.8	4.9 ± 1.1	1.8 ± 0.5
		Digestive	108	8.4 ± 2.6	3.5 ± 0.7	1.6 ± 0.4
**Langstone RSPB**	*n* = 6	Gills	221	12.3 ± 1.5	5.0 ± 0.5	1.2 ± 0.3
		Digestive	259	15.3 ± 2.7	3.6 ± 0.5	2.7 ± 1.4

### Microplastics sizes and shapes in *O. edulis* according to location and tissue

**Fig. 3:**
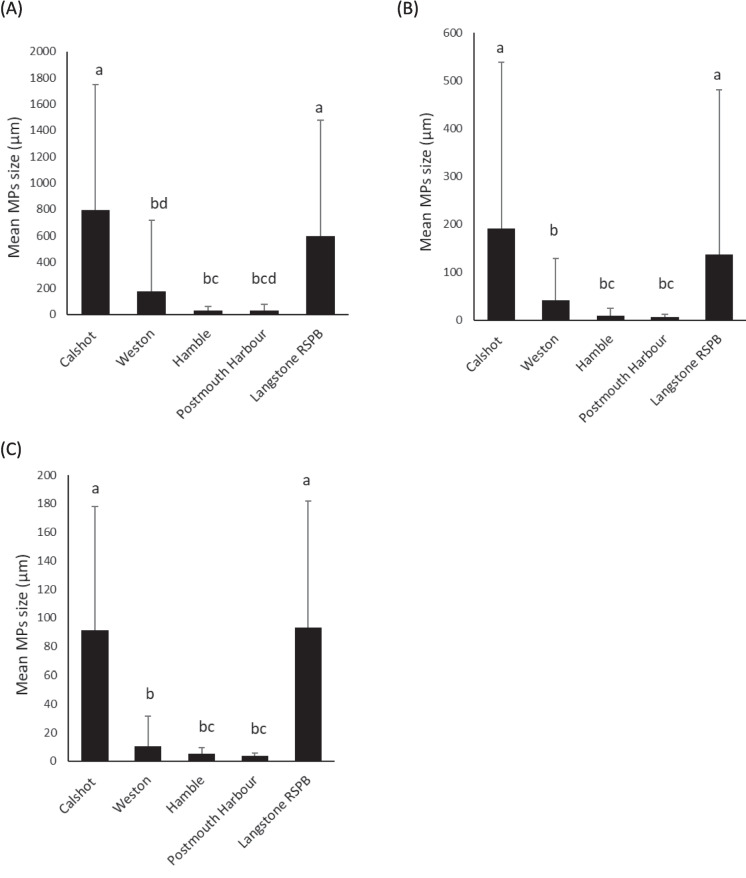
*Ostrea edulis* mean microplastic size for **A** fibres, **B** irregular and **C** round particles collected at Calshot (oysters *n* = 4; MPs per location *n* = 183), Weston (oysters *n* = 5; MPs per location *n* = 221), Hamble (oysters *n* = 8; MPs per location *n* = 626), Portsmouth Harbour (oysters *n* = 4; MPs per location *n* = 241) and Langstone RSPB (oysters *n* = 6; MPs per location *n* = 480). Same letter indicates no significant difference. Error bars represent S.E

Significant differences (*p* < 0.001) between locations were found in particle sizes of the three types (Fig. 3). Overall, plastics from Calshot and Langstone were significantly bigger than those from the other places. Plastics from Hamble and Portsmouth presented similar and smaller sizes regardless of their shape.

**Fig. 4:**
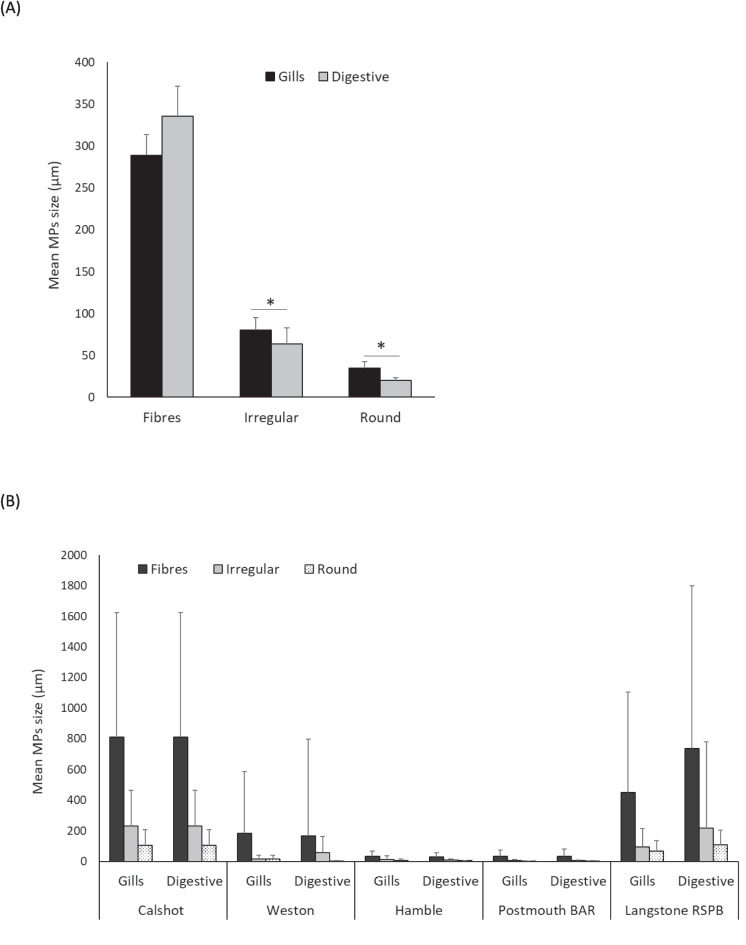
*Ostrea edulis* mean microplastic size **A** in gills and digestive tissue according to their shape (Fibres *n* = 1046; Irregular *n* = 356; Round *n* = 348) and **B** the type of particle (fibres, irregular and round particles) collected at Calshot (Fibres *n* = 108; Irregular *n* = 48; Round *n* = 27), Weston (Fibres *n* = 170; Irregular *n* = 33; Round *n* = 18), Hamble (Fibres *n* = 291; Irregular *n* = 105; Round *n* = 230), Portsmouth Harbour (Fibres *n* = 147; Irregular *n* = 67; Round *n* = 27) and Langstone RSPB (Fibres *n* = 330; Irregular *n* = 103; Round *n* = 46). (*) indicates significant differences. Error bars represent S.E

Microplastic sizes ranged from 0.89 to 6455.91 µm. Hamble and Portsmouth oysters appeared to have the most consistent size ranges per oyster in comparison to the other three locations. Fibres were the largest particles in all the locations (Fig. 3A). Fibres found in oysters from Calshot and Langstone RSPB were larger (790.98 ± 956.53 and 592.22 ± 887.44, respectively) compared to the other locations. Oysters from Calshot and Langstone RSPB also presented larger sizes for irregular and round particles (Fig. 3 B and C). The mean size for MPs found in gills and digestive tissues showed significant differences for irregular (*p* < 0.001) and round particles (*p* < 0.001) with slightly bigger sizes found in gills (Fig. 4A). However, the size observed for fibres, irregular and round particles in both tissues was similar in each location with no significant differences found between tissues (Fig. 4B).

### Effect of oyster size on microplastic size

Oysters collected in this study showed similar biometric parameters (Table S2). Shell widths ranged between 13.87 and 31.71 mm, heights between 52.10 and 93.82 mm and lengths between 51.01 and 106.40 mm. There was an inverse correlation between fibres size and shell length (*r* = − 0.45; *p* = 0.01), height (*r* = − 0.50; *p* < 0.05) and width (*r* = − 0.49; *p* < 0.05). In the same maner, an inverse correlation was found between irregular particle size and shell length (*r* = − 0.47; *p* < 0.05), height (*r* = − 0.48; *p* < 0.05) and width (*r* = − 0.42; *p* < 0.05).

## Discussion

This study records the presence of MPs in wild populations of *O*. *edulis* inhabiting the Solent region. It also shows the presence of these particles in gills and digestive tissue of all oysters analysed.

Fibres were the most common type of particle found in *O*. *edulis*. Fibres as the dominant type of particle ingested by animals is supported by several microplastic studies (Bom & Sá, 2021; Lusher et al., 2013; Thompson et al., 2004). There are a range of potential sources of fibres in the Solent region and the locations showed an important effect on the total amount of particles found. Hamble and Langstone were the locations with more particles found in *O*. *edulis*. As a densely-populated area, there are numerous WWTPs discharging large volumes into the Solent. This is considered an important source, as thousands of fibres pass through the filtering systems of washing machines and are released into wastewater as a result of washing synthetic clothing (Browne et al., 2011; Gallagher et al., 2016). Langstone RSPB Reserve is situated in Langstone Harbour in Portsmouth and is a poorly flushed area with numerous storm-water discharges, and also receives a large discharge of sewage effluent (Soulsby et al., 1978) which makes this area more susceptible to accumulating MPs (Habib et al., 1998; Mahon et al., 2017). This area also receives significant sewage and frequent storm-water discharges, particularly from Budds Farm (Lockwood et al., 1985), causing contaminants to persist and threaten oysters long-term (Oaten et al., 2017).

These characteristics could lead to contaminants remaining near the source and potentially posing a long-term risk to the oysters inhabiting this area. Hamble is a yachting centre of considerable importance and this activity has been related to an increase of MPs in the environment due to the materials and gear used in these activities, such as nets and ropes which are considered significant sources of fibres (Andrady, 2011) and could explain their prevalence in coastal areas. The analysis of trends in time-series data in the Solent has shown that sewage discharges from recreational boats are related to a decrease in marine surface water quality during recreational activities (Xiong et al., 2023).

According to our premise, Calshot was selected as the area perceived to have relatively low levels of pollution. Furthermore, the geographic position of Calshot, near the mouth of the Solent estuary (Williams & Muxagata, 2006) means it is more exposed and experiences greater tidal mixing. Therefore, it is likely that the high flushing rate limits the accumulation of pollutants. Comparatively to the other locations, low population density in the surrounding area, low levels of industrial activity and the absence of any major wastewater discharges in the local vicinity could explain the low numbers of particles found in oysters from this location.

We expected relatively low levels of pollution from terrestrial inputs in Weston. This location is characterised by residential properties and a park-land (Mouchel, 2012). The shoreline consists of shingle and intertidal mudflats and is designated as a Site of Special Scientific Interest (SSSI) (Mouchel, 2012). Weston is located in an open system in the Solent, an ebb-dominant estuary where a greater proportion of water and sediment is transported out of the estuary. Unlike Langstone, MPs are more likely to be transported out of the estuary and away from intertidal sites within the Solent such as Weston, due to its rapid flushing time (Lockwood et al., 1985). However, its proximity to the pollutant inputs from around Southampton City Centre such as from storm-water discharges, Woolston WWTP (Yates, 2015) and other industrial contaminants from the River Itchen including two further WWTPs, shipping and port activity, road debris and a polythene bag and sheet wrapping organisation (Gallagher et al., 2016) mean that the site is still likely to be exposed to a degree of pollution from these sources. These findings support our premise that more populated areas could present greater microplastic quantities, as reported by Andrady (2011).

In this study, irregular MPs were found to be the next most common type of particles, followed by round particles. This tendency has been reported in other studies (Lusher et al., 2013; Manolaki et al., 2023; Tanaka & Takada, 2016). Other studies have found that 88–90% of the MPs items identified in mussel samples were reported as fragments and fibres (Jahromi et al., 2021). The occurrence of irregular MPs is linked to the erosion of synthetic textiles and tire fragments, which are two major sources of microplastic pollution (Boucher & Friot, 2017). The only location where the proportion of round particles was noticeably larger was Hamble. This may be attributable to the proximity of wastewater treatment discharges to these sites which are likely to contain microbeads from cosmetic and personal care products (Boucher & Friot, 2017; Eriksen et al., 2014; Tanaka & Takada, 2016).

It has been reported that damages are determined by the shape of the particles, with non-spherical MPs such as fibres and fragments being more harmful and more toxic than spherical particles (Frydkjær et al., 2017; Rios-Fuster et al., 2023). This means that, even when fibres are the dominant shape of MPs in coastal systems, most ecotoxicological studies and laboratory-based studies focus on spherules and beads effects (Baroja et al., 2021; Khanjani et al., 2023).

The presence of MPs in the digestive system and gills of bivalves has been reported (Van Cauwenberghe & Janssen, 2014; Von Moos et al., 2012). However, no significant difference was found between tissue type and type of microplastic. As the shape of MPs may influence toxicity to the organisms exposed to them (Wright et al., 2013a), this may be an important factor to investigate in future studies, as it could aid the identification of the most harmful MPs and help prioritise which microplastic sources should be managed and minimised.

The concentration of MPs detected in this study was greater than those found in marine bivalves in the literature (Khanjani et al., 2023; Wontor et al., 2023). This result could be a consequence of the varying methods used to digest, filter and identify MPs in the literature (Lusher et al., 2013). For example, most studies have used filters with larger pore sizes, leading to loss of MPs in the smaller size fractions (Li et al., 2015; Thiele et al., 2019; Van Cauwenberghe & Janssen, 2014). The enzymatic protocol used in this study has shown to be an effective method for extracting MPs without destroying any microplastic debris from environmental samples (Cole et al., 2014). Furthermore, the enzymatic digestion method has shown a particle recovery of 97% in environmental samples and animal tissues which makes it an adequate protocol for studies on marine organisms (Karlsson et al., 2017). Even when this method could be more expensive compared to acid digestions, H_2_O_2_, and KOH (Thiele et al., 2019), the results show more efficiency and accuracy leading to more reliable results.

Across the five locations, a variety of different microplastic sizes were found in the oysters. Some of the Langstone oysters were found to have the largest microplastic size ranges, which may be attributable to the diversity of potential sources such as wastewater discharges and pollutants from the surrounding settlements. The MPs observed from Calshot samples have a large range of sizes, despite having comparatively fewer local pollutant sources than the other sites. The area’s proximity to the mouth of the estuary (Williams & Muxagata, 2006) may increase its exposure to a wider range of pollutants sourced from the Solent tributary rivers, the waters around the Isle of Wight and potentially from the Atlantic. The Hamble and Portsmouth oysters, despite being close to several potential MPs sources, were found to have the smallest range of microplastic sizes overall, which could be explained by the dominance of one particular source such as WWTPs over other potential sources. Even though Portsmouth Harbour is a large industrialized estuary with some naval bases and boat centres, it is a natural harbour close to a Special Protection Area and Ramsar site which generates a protected area of ​international importance with minimal human disturbance.

Closer inspection of the round particle sizes from each sampling location revealed that the diameters are not consistent with those reported in the literature. Due to their uniform, spherical shape, sizes and colouring, the particles were suspected to be microbeads. However, they do not appear to fit the size profile of the cosmetic microbeads, stated to be > 0.1 mm in size and they could potentially be nanoplastics-particles < 1 µm (Hartmann et al., 2019). The colouring of these beads could be indicative of photo-oxidative damage, meaning the particle has persisted in the marine environment for a long period (Acosta-Coley & Olivero-Verbel, 2015).

As microplastic sizes get smaller over time, their surface areas increase, and their potential to adsorb organic compounds (OCs) from natural waters and contain them on a solid surface, making them more bioavailable to some organisms (Costigan et al., 2022; Fu et al., 2021). It has been reported that the relative size of particles (area and perimeter) could be the most important factor in predicting whether a MP will be rejected or ingested by bivalves (Mladinich et al., 2022). Other studies have shown that larger particles may be retained in the guts of bivalves for shorter periods than smaller particles (Bouwmeester et al., 2015; Lusher et al., 2013) and that smaller MPs are more susceptible to translocation into the circulatory system (Browne et al., 2008; Jahromi et al., 2021; Van Cauwenberghe et al., 2015; Von Moos et al., 2012). It has been suggested that bivalves could take the particles via the mouth, transported to the gastrointestinal tract and internalised into cells of the digestive system by endocytosis (Von Moos et al., 2012). Some features such as shape, surface properties, chemical composition, and particle ageing are of toxicological importance and a fundamental issue for future ecotoxicological research (Rozman & Kalčíková, 2022).

The occurrence of MPs within bivalves is likely a result of ingestion. Oysters, as filter feeders, exhibit a filtration rate of approximately 200 L of water a day (Harding et al., 2016). During their feeding process, these organisms sieve water through their inhalant siphon, allowing it to pass over their complex folded gills, where they selectively target plankton and other food particles, including microscopic-sized MPs. This behaviour potentially increases their inadvertent or deliberate ingestion of MPs. Consequently, oysters can passively consume MPs through filter feeding, either unintentionally or by actively selecting and consuming sinking MPs from the surrounding waters. For this reason, a higher concentration of particles in the digestive tissue compared to the gills could be expected, as has been observed in *Magallana gigas* (formerly *Crassostrea gigas*) (Sussarellu et al., 2016). However, our study did not find the same trend in *O*. *edulis*.

Contradictory results regarding the differences between tissues have been reported. Some studies have shown that the gills could present higher concentrations of MPs in *Crassostrea virginica* in comparison to digestive tissue (Wontor et al., 2023). Other studies have reported higher numbers or concentrations of MPs in digestive tissue of *Meretrix lyrate, Meretrix meretrix*, *Crassostrea gigas*, *Anadara granosa* and *Mactra grandis* (Doan et al., 2023). In this study, the values obtained for MPs in the gills of oysters collected at different locations were similar. However, the values of MPs concentrations in digestive tissue showed greater variations between locations. A combination of several tissues or whole organisms should be included in microplastic studies in bivalves given that the dynamics related to the particular dynamics of the tissues, translocation and elimination processes in bivalves are not yet understood. Further research is needed to determine whether an ingestion and/or translocation of MPs play an important role in the distribution of these particles in *O*. *edulis*.

It was expected that oysters with larger shells, filtering larger volumes of water are more likely to uptake greater quantities of MPs. However, comparisons of oyster shell length and mean microplastic count suggested an inverse relationship between these two factors. Two atypically large specimens were found to have among the lowest mean microplastic counts. The largest oyster had, by far, the lowest microplastic count overall. The same lack of correlation bwteeen average size of and shell size has been reported by other studies (Wu et al., 2022). As shell length is an indicator of age (Ridgway et al., 2011), this may suggest that *O*. *edulis* does not retain all MPs it intakes throughout its life and may release them into the surrounding environment. One of the functions of the gills in bivalves is for the sorting and rejection of unwanted filtered material as pseudofaeces or faeces (Ward et al., 2019; Xu et al., 2017). The production of pseudofaeces could work as a mechanism that limits the retention of MPs within *Atactodea striata*, *Crassostrea virginica* and *Mytilus edulis*, and may explain why quantities of MPs are not necessarily proportional to oyster size (Ward et al., 2019; Xu et al., 2017). In addition, smaller (younger) shellfish with elevated metabolic demands may filter larger volumes of seawater, as they have a greater likelihood of accumulating a higher quantity of MPs from the surrounding environment (Chinfak et al., 2021). Further studies using larger sample sizes are required to determine whether microplastic loadings are related to *O*. *edulis*’ size and whether they change during the species’ life cycle.

## Conclusions

To the best of our knowledge, this is the first study to investigate the presence of MPs in wild populations of *O*. *edulis*. The detection of MPs in wild *O*. *edulis* suggests the potential accumulation of these particles in filter-feeding organisms. MPs are clearly present within *O*. *edulis* populations in the Solent region. MPs were detected in gills and digestive tissues of oysters from all locations with no significant difference between tissues concerning the amount, concentration, or size of MPs. However, the quantities of MPs found in the oysters differ depending on the sampling location. These findings support our premise that more populated areas could present greater microplastic quantities. The comparison between the sampling sites revealed that oysters from Langstone contained significantly larger quantities of MPs. Conversely, the Calshot oysters contained the smallest quantities. This was an unsurprising discovery as Langstone harbour is poorly flushed and affected by a plethora of pollutant sources discharging into the area, whereas Calshot is surrounded exposed to greater tidal mixing and surrounded by a less polluted area. In agreement with the literature, fibres were found to be the commonest microplastic type in this study. The prevalence of fibres and fragments observed in bivalves samples might result from elevated laundry discharge, numerous WWTPs, storm-water discharges associated with the densely-populated settlements, commercial shipping and fisheries and recreational sailing in the Solent Region. *O*. *edulis* could be a useful tool in monitoring studies to evaluate marine microplastic pollution. The wide range of MPs accumulation and frequency in bivalves emphasizes the need for more comprehensive studies to understand the extent of MPs contamination and its potential ecological and human health impacts. This might influence policymaking regarding plastic production, addressing a significant environmental concern.

## Supplementary Information

Below is the link to the electronic supplementary material.

Supplementary file1 (DOCX 5446 KB)

Supplementary file2 (DOCX 148 KB)

Supplementary file3 (DOCX 15 KB)

Supplementary file4 (DOCX 16 KB)

## Data Availability

Data will be placed in the University of Southampton repository if and when the paper advances to the next phase.
