# Supplementary material for: Microplastics in the European native oyster, Ostrea edulis, to monitoring pollution-related patterns in the Solent region (United Kingdom)
Source: Environ Monit Assess. 2025 Apr 12;197(5):544. doi: 10.1007/s10661-025-13975-x (PMC11993462; doi:10.1007/s10661-025-13975-x)
Supplement: Supplementary file 1 — Supplementary file1 (DOCX 5446 KB) [file 10661_2025_13975_MOESM1_ESM.docx]

**B**

**A**


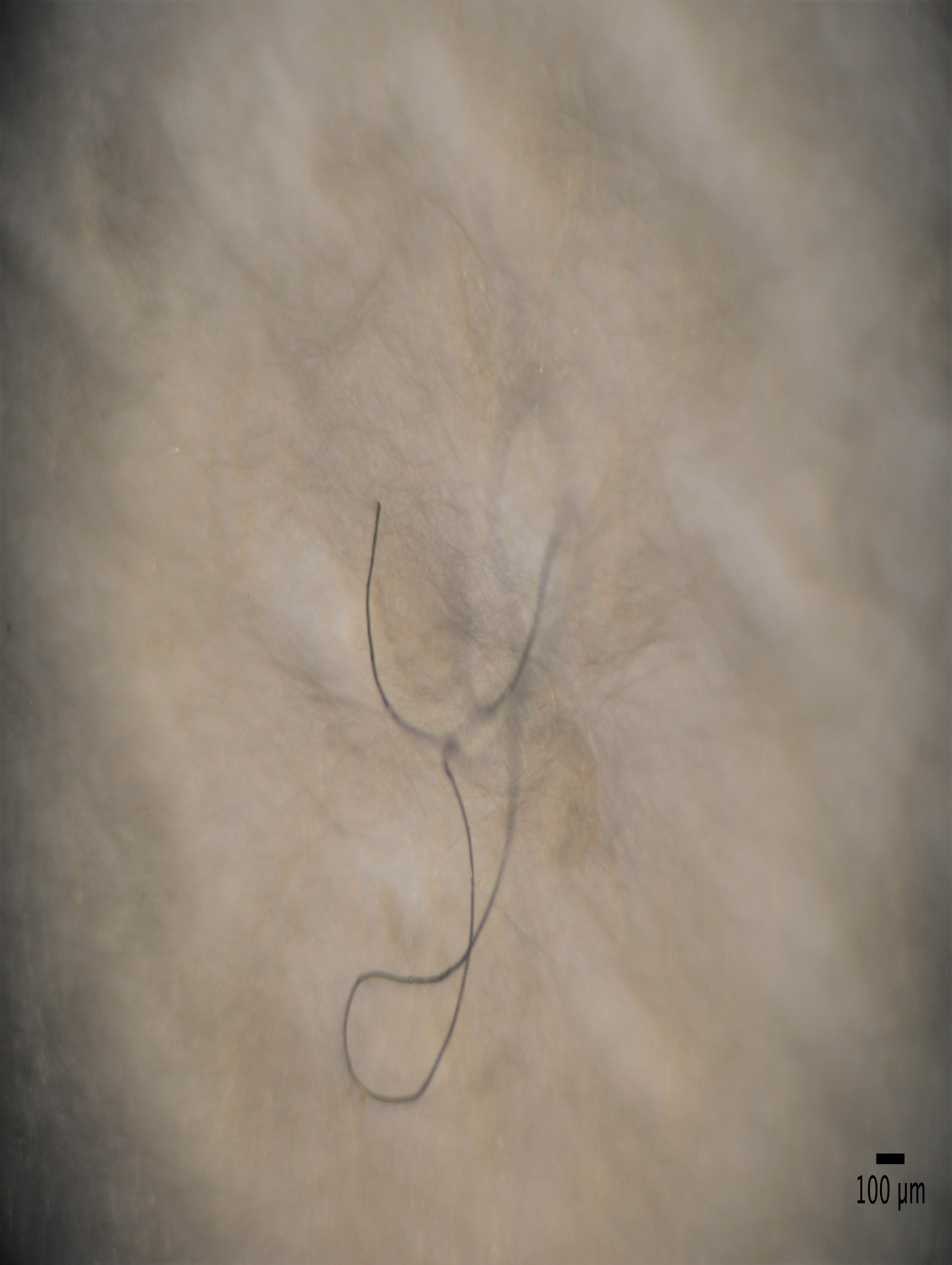


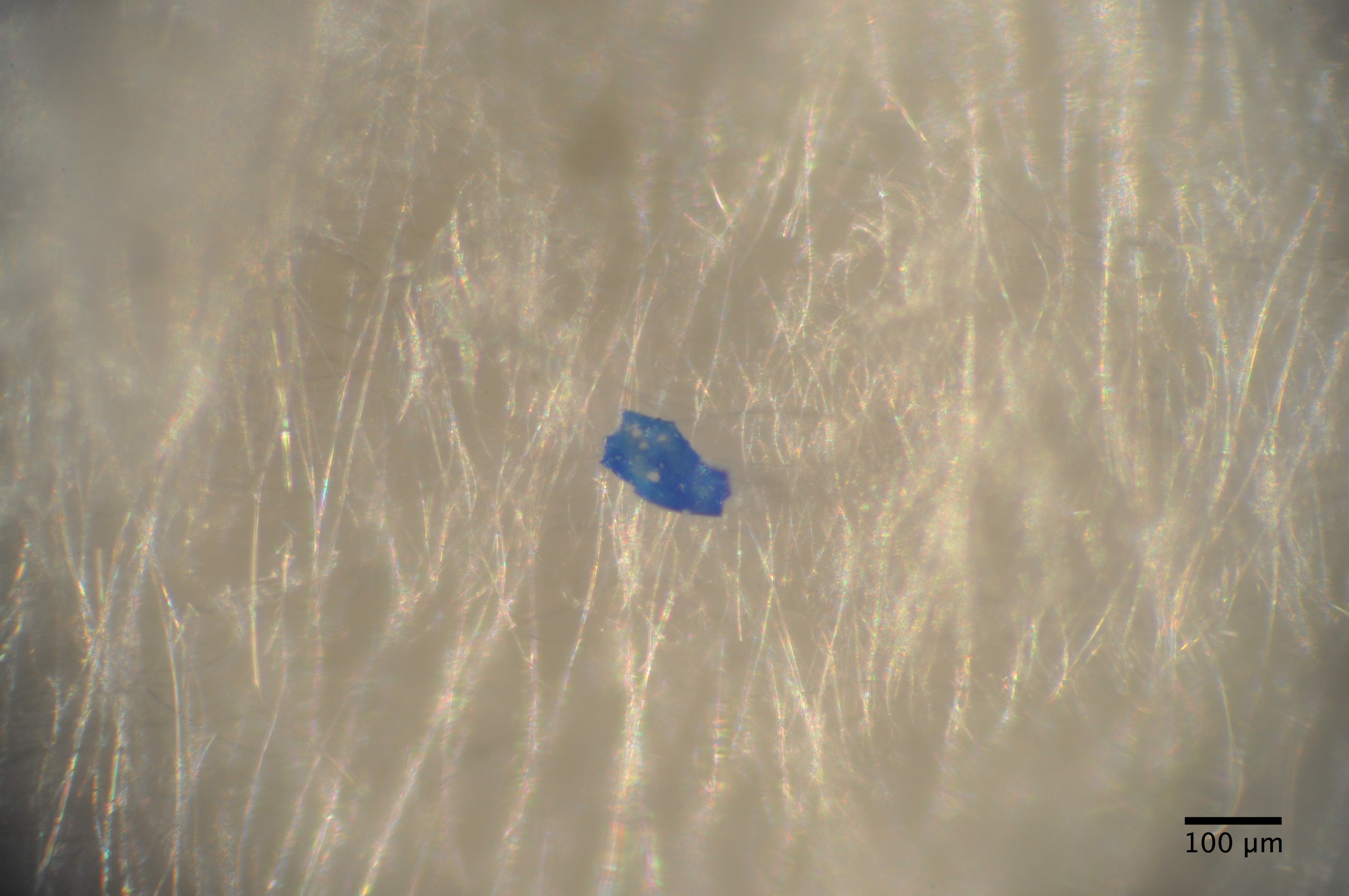


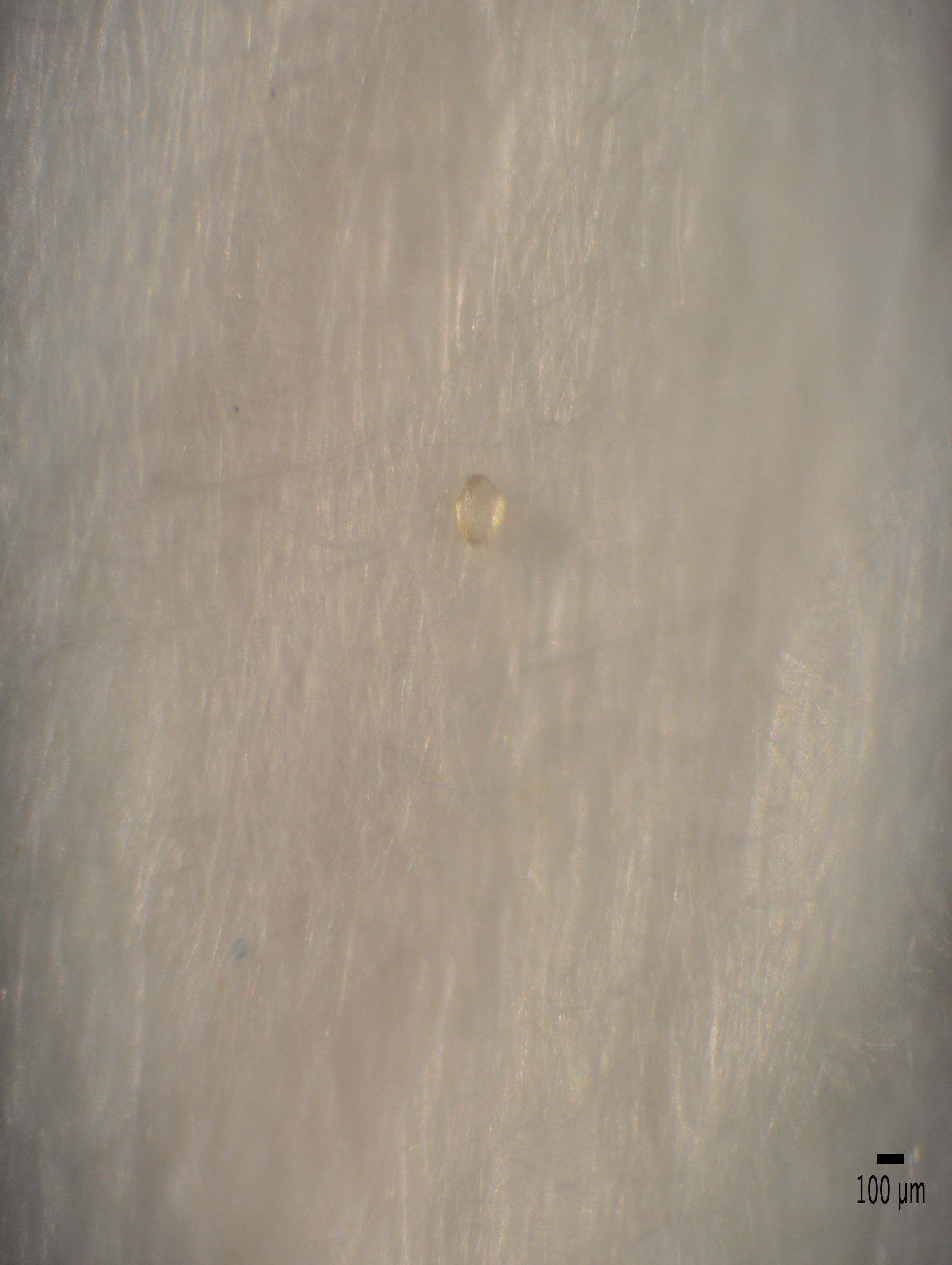


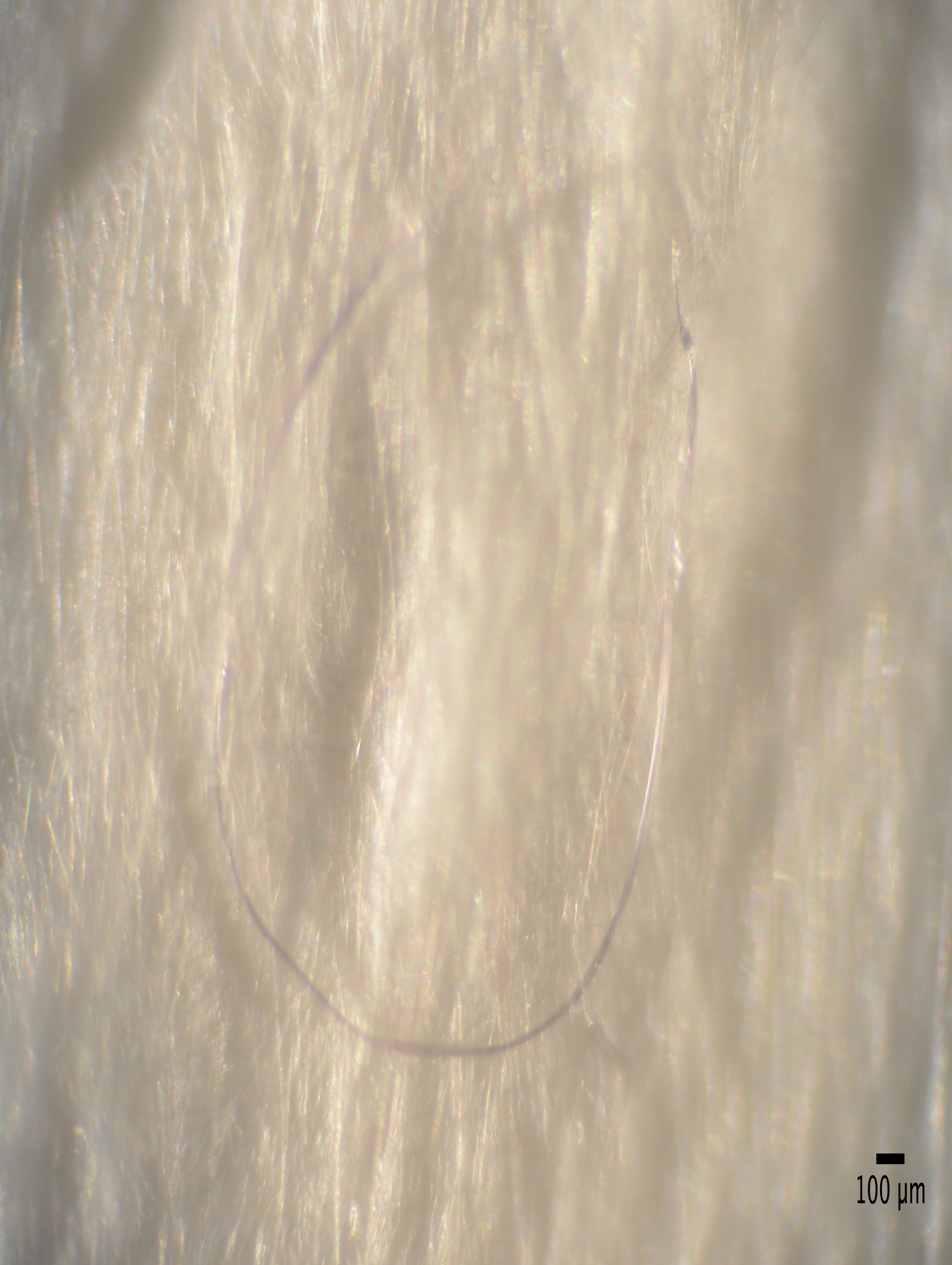


**D**

**C**

**Supplement, Figure 1.** Photographs showing examples of the different types of microplastics found in *O. edulis* tissue extracts*.* Examples of fibres (A, D), an irregular microplastic (B) and a round microplastic (C) are shown. All photographs (A-D) were captured directly on the filter paper. A and D also illustrate two of the most common colours of fibres observed in the *O.edulis* samples. Scale bar = 100μm.
