# Supplementary material for: Microplastics in the European native oyster, Ostrea edulis, to monitoring pollution-related patterns in the Solent region (United Kingdom)
Source: Environ Monit Assess. 2025 Apr 12;197(5):544. doi: 10.1007/s10661-025-13975-x (PMC11993462; doi:10.1007/s10661-025-13975-x)
Supplement: Supplementary file 2 — Supplementary file2 (DOCX 148 KB) [file 10661_2025_13975_MOESM2_ESM.docx]

**
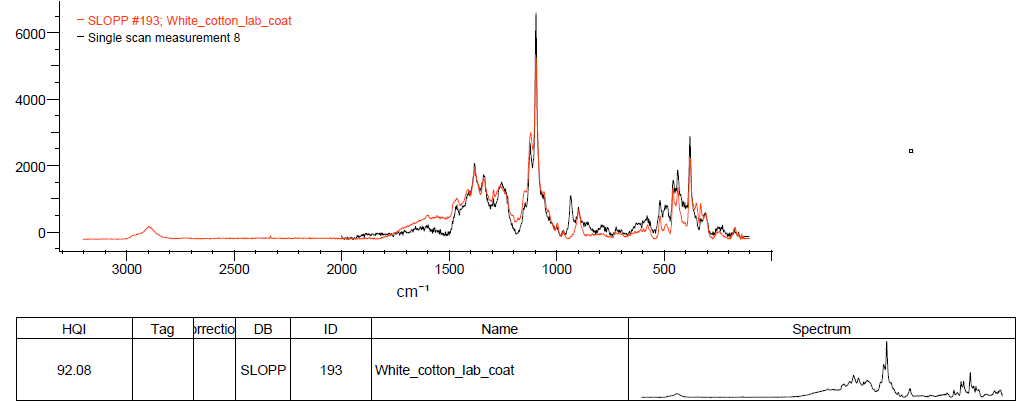
**

**
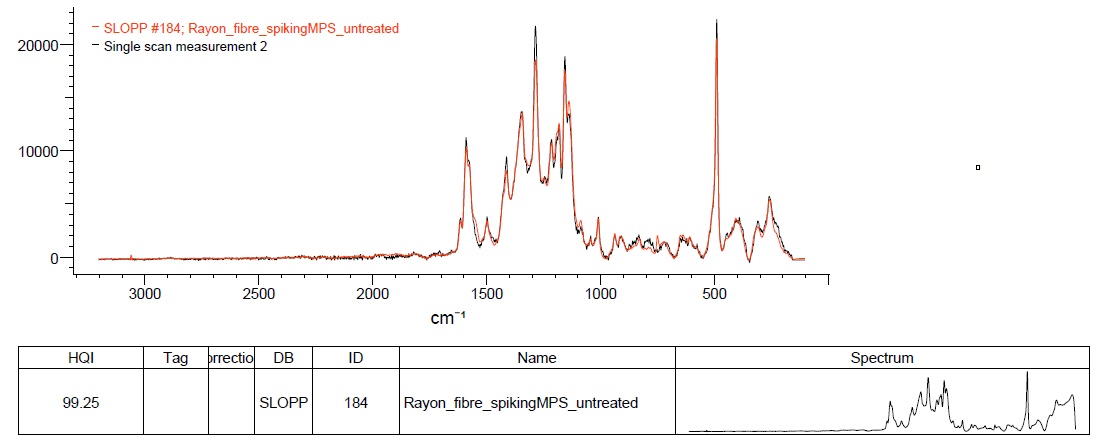
**

**
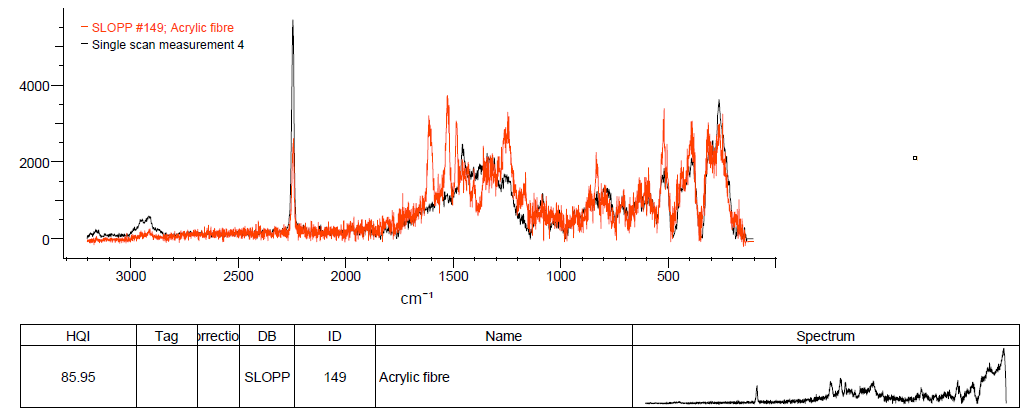
**

**Supplement, Figure 2.** Examples of spectra to confirm the identity of particles identified as microplastics
