# Supplementary material for: Microplastics in the European native oyster, Ostrea edulis, to monitoring pollution-related patterns in the Solent region (United Kingdom)
Source: Environ Monit Assess. 2025 Apr 12;197(5):544. doi: 10.1007/s10661-025-13975-x (PMC11993462; doi:10.1007/s10661-025-13975-x)
Supplement: Supplementary file 3 — Supplementary file3 (DOCX 15 KB) [file 10661_2025_13975_MOESM3_ESM.docx]

**Table S1.** Colour classification to standardize criteria and define categories for this study

|  | Colour classification | |
| --- | --- | --- |
| Type of microplastic | Colours | Included colour variations (if any) |
| Fibre | Blue | Medium, light and dark blue |
|  | Black |  |
|  | Orange | Dark, amber |
|  | White | Clear-white, opaque-white |
|  | Pink |  |
|  | Green | Light green, dark green, green-brown |
|  | Yellow | Clear-yellow |
|  | Red | Clear-red |
|  | Purple | Dark purple, lavender |
|  | Brown | Dark, medium |
|  | Grey | Dark, medium, light |
| Irregular | Black |  |
|  | Green | Dark, medium |
|  | Orange | Amber |
|  | White | Transparent-white, opaque-white |
|  | Blue | Dark, medium, light |
|  | Red |  |
|  | Grey | Dark, light |
| Round | Blue | Dark, medium, light |
|  | Grey |  |
|  | Black |  |
|  | Yellow | Transparent-yellow, brown-yellow |
|  | White | Transparent-white, opaque-white |
