# Supplementary material for: Microplastics in the European native oyster, Ostrea edulis, to monitoring pollution-related patterns in the Solent region (United Kingdom)
Source: Environ Monit Assess. 2025 Apr 12;197(5):544. doi: 10.1007/s10661-025-13975-x (PMC11993462; doi:10.1007/s10661-025-13975-x)
Supplement: Supplementary file 4 — Supplementary file4 (DOCX 16 KB) [file 10661_2025_13975_MOESM4_ESM.docx]

**Table S2.** Biometric parameters measured in oysters collected at each location

| **Location** | **ID** | **Shell length (mm)** | **Shell height (mm)** | **Shell width (mm)** |
| --- | --- | --- | --- | --- |
| Calshot | Ca1 | 60.30 | 60.99 | 17.28 |
|  | Ca2 | 55.95 | 64.87 | 20.57 |
|  | Ca3 | 68.19 | 71.04 | 15.22 |
|  | Ca4 | 51.01 | 52.10 | 16.54 |
| Weston | We1 | 55.01 | 56.88 | 21.11 |
|  | We2 | 83.54 | 93.82 | 31.71 |
|  | We3 | 53.83 | 58.82 | 17.85 |
|  | We4 | 54.93 | 65.33 | 18.16 |
|  | We5 | 55.49 | 65.42 | 16.23 |
| Hamble | Ha1 | 57.65 | 63.54 | 22.91 |
|  | Ha2 | 59.51 | 67.92 | 17.09 |
|  | Ha3 | 86.45 | 81.81 | 22.44 |
|  | Ha4 | 60.82 | 71.02 | 21.50 |
|  | Ha5 | 83.10 | 84.20 | 23.90 |
|  | Ha6 | 99.60 | 88.90 | 29.60 |
|  | Ha7 | 97.70 | 81.80 | 24.40 |
|  | Ha8 | 86.40 | 83.50 | 22.20 |
| Langstone RSPB | La1 | 63.45 | 67.43 | 18.55 |
|  | La2 | 67.12 | 61.86 | 14.09 |
|  | La3 | 53.77 | 65.76 | 19.59 |
|  | La4 | 56.64 | 67.36 | 18.59 |
|  | La5 | 65.23 | 71.50 | 20.50 |
|  | La6 | 66.32 | 60.56 | 13.87 |
| Portsmouth Harbour | BAR1 | 82.00 | 71.70 | 20.50 |
|  | BAR2 | 69.90 | 68.20 | 20.40 |
|  | BAR3 | 88.20 | 91.00 | 29.70 |
|  | BAR4 | 106.40 | 80.40 | 26.00 |
